# Malignant mesothelioma

**DOI:** 10.12669/pjms.296.3938

**Published:** 2013

**Authors:** Ishtiaq Ahmed, Salman Ahmed Tipu, Sundas Ishtiaq

**Affiliations:** 1 Prof. Dr. Ishtiaq Ahmed, Professor, Department of Surgery, Al-Nafees Medical College, Islamabad, Pakistan.; 2 Dr. Salman Ahmed Tipu, Assistant Professor of Surgery, Al-Nafees Medical College, Islamabad, Pakistan.; 3 Sundas Ishtiaq, Women Medical College, Abbotabad, Pakistan.

**Keywords:** Asbestos, Malignant Mesothelioma, Peritoneum, Surgery

## Abstract

Malignant Mesothelioma (MM) is a rare but rapidly fatal and aggressive tumor of the pleura and peritoneum with limited knowledge of its natural history. The incidence has increased in the past two decades but still it is a rare tumor. Etiology of all forms of mesothelioma is strongly associated with industrial pollutants, of which asbestos is the principal carcinogen. Mesothelioma is an insidious neoplasm arising from mesothelial surfaces i.e., pleura (65%-70%), peritoneum (30%), tunica vaginalis testis, and pericardium (1%-2%). The diagnosis of peritoneal and Pleural mesothelioma is often delayed, due to a long latent period between onset and symptoms and the common and nonspecific clinical presentation. The definite diagnosis can only be established by diagnostic laparoscopy or open surgery along with biopsy to obtain histological examination and immunocytochemical analysis. Different treatment options are available but Surgery can achieve a complete or incomplete resection and Radical resection is the preferred treatment. Chemotherapy has an important role in palliative treatment. Photodynamic therapy is also an option under trial. Patients who successfully underwent surgical resection had a considerably longer median survival as well as a significantly higher 5-year survival.

***Source of Data/Study Selection:*** The data were collected from case reports, cross-sectional studies, Open-label studies and phase –II trials between 1973-2012.

***Data Extraction:*** Web sites and other online resources of American college of surgeons, Medline, NCBI and Medscape resource centers were used to extract data.

***Conclusion:*** Malignant Mesothelioma (MM) is a rare but rapidly fatal and aggressive tumor with limited knowledge of its natural history. The diagnosis of peritoneal and Pleural mesothelioma is often delayed, so level of index of suspicion must be kept high.

## INTRODUCTION

Malignant Mesothelioma (MM) is a rare but rapidly fatal and aggressive tumor of the pleura and peritoneum with limited knowledge of its natural history. The earliest mention of a possible tumor of the chest wall (the pleura) was made in 1767 by Joseph Lieutaud, the founder of pathologic anatomy in France in study of 3,000 autopsies where he found two cases of “pleural tumors.” In 1819, René-Théophile-Hyacinthe Laennec, the French physician based upon his understanding of the nature of pleural cells, suggested the origin from the pleura. Peritoneal mesothelioma was first described in 1908 by Miller and Wynn.^1^^,^^2^ The incidence has increased in the past two decades but still it is a rare tumour.^1^^,^^3^ The disease incidence varies geographically i.e., from less than 1 per 1,000,000 in Tunisia and Morocco, to the highest rate in Britain, Australia and Belgium i.e. 30 per 1,000,000 per year.^3^ Currently, the incidence ranges from about 7 to 40 per 1,000,000 in industrialized Western nations, depending upon the amount of asbestos exposure in the past several decades.^4^ Occupational and environmental asbestos exposure still remains a major causative factor. It is more common in male and the risk increases with age, but this disease can appear at any age in either sex. Incidence of Malignant Peritoneal Mesothelioma (MPM) is less as compared to pleural mesothelioma and only 20% to 33% of all mesotheliomas (one fifth to one third) arise from the peritoneum.^5^^,^^6^ The diagnosis of peritoneal mesothelioma is often delayed, due to a long latent period between onset and symptoms and the common and non specific clinical presentation. Due to its unusual nature, the disease has not been clearly defined in terms of its natural history, diagnosis or management. Moreover, the treatment options are far from satisfactory. Moreover, because malignant peritoneal mesothelioma (MPM) usually remains confined to the peritoneal cavity during most of its natural history, so cytoreductive regional chemotherapy is an attractive option in treatment.^7^ Because of lack of effective treatment, median survival time is 5 to 12 months only and mean symptoms-to-survival time is 345 days.^2^^,^^6^

## EPIDEMIOLOGY

Mesothelioma is an insidious neoplasm arising from mesothelial surfaces i.e., pleura (65%-70%), peritoneum (30%), tunica vaginalis testis, and pericardium (1%-2%).^1^ In the United States, the overall prevalence is 1-2 cases per million or 3300 cases every year from which only 10 to 15 percent (200 -400 new cases annually) are peritoneal mesothelioma.^8^ In the USA, incidence rates of Malignant Peritoneal Mesothelioma have remained stable over the last 30 years.^8^^,^^9^ Generally, all over the world the incidence of peritoneal mesothelioma is one order of magnitude lower than pleural mesothelioma.^5^ Incidence is rising worldwide due to increase occupational asbestos exposure. The annual incidence of MM was approximately 1.00 cases per 100,000 in 2005.^9^ Overall, incidence of mesotheloma is expected to peak between 2015 and 2025 and among them the pleural mesothelioma accounts for most of the rising number of cases. Primary malignant mesothelioma is a highly aggressive malignancy that occurs most commonly in older men and that has a strong association with high levels of occupational asbestos exposure.^10^^,^^11^ It is more common in male and the incidence of peritoneal mesothelioma is 0.5–3.0 per million per year in male, and 0.2–2.0 per million per year in female.^1^^,^^5^ The female component was particularly high for EPMM, with male/female ratios of 1.4:1 and 1.9:1 for peritoneal and pericardium, respectively, and there were 2.7 male pleural MM cases for each female in ReNaM, in agreement with other published case lists.^5^ It can occur in any age group but more common in 6^th^ decade and only about 2% to 5% of all cases reported in the first two decades of life.^12^

Aetiology of all forms of mesothelioma is strongly associated with industrial pollutants, of which asbestos is the principal carcinogen. Among Asbestos (primarily the crocidolite variety) is considered the main risk factor. Other risk factors in some patients are radiation exposure, talc, erionite or mica exposure, and patients suffering from familial Mediterranean fever and diffuse lymphocytic lymphoma. Literature review shows that only 50% of patients with a peritoneal origin of MPM have a history of asbestos exposure.^5^^,^^9^

## PATHOGENESIS

Regarding pathogenesis, the asbestos fibers are ingested and work their way from digestive organs into the peritoneal membrane or these fibers are inhaled and travel to the peritoneal membrane via the lymphatic system. After entering the peritoneal layers they get trapped and cause changes in mesothelial cells, leading to irritation and inflammation. The exact way in which asbestos fibers cause these changes is uncertain, but researchers believe such changes are responsible for cancer development.^13^^,^^14^

Most malignant mesotheliomas have complex karyotypes with extensive aneuploidy and the rearrangement of many chromosomes. The single most common karyotypic change found is the loss of one copy of chromosome 22. Other chromosomal changes commonly observed are deletions in the chromosome arms 1p, 3p, 9p, and 6q. Several changes in the tumor suppressor genes p16 (*CDKN2A*) and p14 (*ARF*) and loss of function of neurofibromin-2 (NF2) have also been noted.^15^

Mesothelioma can arise from both visceral and parietal peritoneum. Grossly it appears most often as diffuse sheetlike or nodular thickening of the peritoneal surfaces and occasionally as a localized mass over the peritoneal surfaces. Multicystic mesothelioma occurs most commonly in female and has benign or indolent biologic behavior in the majority of patients. Multilocular cystic type arises from the pelvic peritoneal surfaces. Primary peritoneal serous carcinoma occurs almost exclusively in female and is histologically identical to ovarian serous carcinoma. It may be indistinguishable from metastatic ovarian carcinoma at imaging studies.^11^^,^^16^

## CLINICAL FEATURES AND DIAGNOSIS

Clinical symptoms are usually atypical and nonspecific. Commonest initial symptoms are pain (35%), distension (31%) of abdomen, less frequently night sweats but they often develop cachexia, weight loss (69%), anorexia or ascites (77%).^7^^,^^12^^,^^17^ Rarely, patient presents with fever of unknown origin, intestinal obstruction or acute abdominal surgical emergency.^17^^-^^19^ Occasionally, it is an incidental diagnosis during laparoscopy. Paraneoplastic syndromes particularly associated with peritoneal mesothelioma are thrombocytosis, hypercoagulability, hypoglycemia, venous thrombosis, paraneoplastic hepatopathy, and a wasting syndrome.^1^^,^^12^ Majority of patient report late due to very elusive and atypical symptoms which leads to its diagnosis in advanced stages. Moreover, usually it takes a considerable time to reach the correct diagnosis and statistically mean symptoms-to-diagnosis time reported is about 122 days.^1^ Routine laboratory tests and abdominal radiograph are not helpful in making the precise diagnosis.

Ultrasound scan and Computed tomography (CT) findings are also insufficient and nonspecific to establish a diagnosis of peritoneal mesothelioma. However, CT is useful in detection, localization, staging and in guiding biopsy of peritoneal masses.^20^ Peritoneal masses appear as heterogeneous, solid and enhancing soft-tissue masses on CT showing expansive rather than infiltrating growth pattern.^16^ Mainly three types of appearances are described on CT scan. The most common is 'Dry-painful' type, in which a large mass or multiple small peritoneal masses in abdominal quadrants, with no signs of ascites are seen. The 'wet' type, is associated with ascities and gut distension along with widespread small nodules and plaques, without any solid masses. The 'mixed' type is having features of both dry and wet types.^21^ Literature review shows insufficient data about the sonographic and MRI manifestations of peritoneal mesothelioma. However, CT scan and Ultrasonography are helpful in providing important information during the diagnostic process.

***Diagnostic laparoscopy:*** Due to nonspecific clinical and radiologic presentation of mesothelioma, the definite diagnosis can only be established by diagnostic laparoscopy or open surgery along with biopsy to obtain histological examination and immunocytochemical analysis. The importance of diagnostic laparoscopy in the diagnosis of peritoneal mesothelioma is usually overlooked by other diagnostic modalities, such as ultrasound, Computed tomography and ascitic fluid cytology. Literature reports that laparoscopy can lead to dissemination of tumour to port sites and may complicate the situation.^7^

***Role of cytology/histology:*** The distinction between a benign and malignant tumour and a primary from the metastatic one is another main challenge other than diagnosis. Therefore, the histological and immunohistochemical examination have important role in definitive diagnosis of peritoneal mesothelioma. Ascitic fluid cytology has a low diagnostic potential, due to high cytologic diversity of tumour and small number of malignant cells within the fluid. In dry type of PM, fine-needle aspiration of the tumour can be used for diagnosis. Cytological markers in Immunohistochemistry used to determine whether the tissue is mesothelial in origin are Calretinin and Wilms' Tumor 1 antigen (WT1), whereas the Epithelial Membrane Antigen (EMA) is used to determine whether the tissue is malignant or not. Potentially useful serum markers for diagnosis and follow-up are the serum mesothelin-related protein (SMRP), which is elevated in more than 84% of mesotheliomas, and has a 60% sensitivity at diagnosis. Inaddition, CA-125, CA 15-3, hyaluronic acid, and osteopontin^22^ are other potential markers in the diagnosis of PM. Studies show that, a positive immunostain for calretinin has markedly increased the accuracy of diagnosis.^23^ The malignant peritoneal mesothelioma can be diagnosed upto 80% of cases with an adequate cytologic sample and by experienced cytologists.^4^

Immunohistochemistry is also useful in distinguishing peritoneal mesotheliomas from primary papillary serous carcinoma of peritoneum, serous ovarian carcinomas, colorectal adenocarcinoma diffusely involving the peritoneum and borderline serous tumors. In particular, calretinin, cytokeratin, and thrombomodulin are typically positive in patients with mesotheliomas and negative in serous carcinomas.^24^

In the absence of ascities or in case of inconclusive cytology, tumor biopsy should be done to reach a diagnosis. Diagnostic accuracy increases with core sample size because the immunohistochemical expression of tumor markers is not homogeneous within the same solid tumor section.

## TREATMENT

Surgery can achieve a complete or incomplete resection and Radical resection is the preferred treatment in malignant peritoneal mesotheliomais which is associated with better prognoses and should be pursued when possible.^9^^,^^24^ Other treatments comprises of intensive loco-regional therapeutic strategies such as cytoreductive surgery, hyperthermic intraoperative or early postoperative intraperitoneal chemotherapy and immunotherapy.

Radical resection is often not possible so the other alternative and better option is cytoreductive surgery which is aimed to remove as much tumor as possible.^10^ The surgical debulking is classified according to the Completeness of Cytoreduction Score which is also widely used in both invasive and non invasive peritoneal surface malignancies. This comprises of the evaluation of residual peritoneal seeding within the operative field and is thought to be the principle prognostic indicator. Completeness of Cytoreduction Score comprises of complete cytoreduction (CC-0) or partial with a diameter of the residual nodules < 0.25 cm (CC-1), 0.25-2.5 cm (CC-2) and > 2.5 cm or confluence of tumor nodules (CC-3).^24^^,^^25^ The CC-1 tumor is considered penetrable by intracavitary chemotherapy and is, therefore, designated as complete cytoreduction if perioperative intraperitoneal chemotherapy is used. The limitation of this score is that it can be evaluated only after surgery therefore; no preoperative assessment about resectability of the tumor can be done.^25^ Mean Survival after cytoreductive surgery and intraperitoneal chemotherapy is 35.8 months in CC-0 or CC-1 resection, and only 6.5 months with a CC-2 or CC-3 resection.^25^

Chemotherapy, intraperitoneally or systemically has an important role in palliative treatment. Direct exposure of antitumor agent to the peritoneal surface is considered to be most effective against malignant peritoneal mesothelioma. Literature review shows that the overall response rate with a single agent, combined, intraperitoneal chemotherapy and continuous hyperthermic peritoneal perfusion are 13.1%, 20.5%, 47.4%, and 84.6%, respectively.^26^ Intraperitoneal chemotherapy can be instilled without surgery through an abdominal catheter or after surgery. Advantages of intraperitoneal chemotherapy include less systemic toxicity and greatly enhanced drug concentrations in the peritoneal cavity.^27^ Continuous intraperitoneal hyperthermic perfusion after cytoreductive surgery for resectable tumors is the standard treatment at diagnosis.^24^^,^^28^^-^^30^ In this a preheated (42.5 degrees C) perfusate with 2 or 3 antineoplastic agents (i.e. Cisplatin, Mitomycin C, Fluorouracil, Doxorubicin, and/or Paclitaxel) is continuously infused post operatively into the closed or semi-closed abdomen. Up to 12% of major morbidity is reported in literature and the most significant complications are anastomotic leaks (11%), abdominal bleeds (1.9%) and sepsis (1.9%).^24^^,^^30^ About 12% operative mortality is also reported. Cisplatin is the most studied drug, with activity in 25% of patients.^27^

Regarding systemic chemotherapy literature shows that the Pemetrexed in combination with cisplatin has improved survival in patients with peritoneal mesothelioma as in pleural mesothelioma.^31^^,^^32^ Data from uncontrolled studies recommends it as a standard of care for patients with unresectable malignant mesothelioma. About 71.2% of the disease control rate reported which includes partial responses or stable disease. Complete responses are not reported from this chemotherapy.^33^ Other regimens like Vinorelbine and Gemcitabine, either alone or combined with platinum compounds are also used for unresectable disease.^32^ Among them the response rate of Vinorelbine alone is 24%^34^ whereas the Irinotecan^35^ and Gefinitib^36^ have not proved effective when used alone. Overall survival of 17 and 92 months (70%), 5-year overall survival, 63%, disease-free survival, and 51% progression-free survival after cytoreductive surgery and continuous hyperthermic peritoneal perfusion with Cisplatin, Fluorouracil, and Paclitaxel reported in literature.^28^

Over the past decade, the management of peritoneal mesothelioma has evolved similarly to ovarian cancer treatment i.e. cytoreductive surgery, heated intraoperative intraperitoneal chemotherapy (HIIC) with cisplatin, doxorubicin, and early postoperative intraperitoneal paclitaxel. These perioperative treatments are followed by adjuvant intraperitoneal Paclitaxel and second-look cytoreduction. This multimodality treatment i.e. cytoreductive surgery and intraperitoneal chemotherapy has resulted in a median survival of 50 to 60 months.^21^^,^^23^

Radiotherapy has a very limited role in treatment of peritoneal mesothelioma, so it is not currently used.^37^

Immunotherapy is being used on an experimental basis at present, mainly for pleural mesothelioma.^38^ Mainly, the Humanized anti-CD3 antibodies (OKT3), Cytotoxic T lymphocytes (CTL), Interferon alfa-2a, and autovaccine have been used anecdotally for palliation of advanced peritoneal mesothelioma, but further studies are needed before this therapy can be recommended.^38^^-^^40^

Gene therapy is currently another exploring way by the genetic researchers to safely treat mesothelioma patients. Suicide gene therapy is one of the most promising forms of gene therapy for the treatment of mesothelioma. Clinical trials conducted at the University of Pennsylvania Medical Center shows that the suicide gene therapy is effective in reducing the size and severity among four of the 34 patients studied. Another type of gene therapy is under trial in which modified viruses are used to deliver immune system molecules called cytokines which control and direct immune response. When introduced through gene therapy, these cytokines can help the immune system to mount an attack against cancer cells.^41^^,^^42^

Photodynamic therapy is also an option under trial in which medical researchers seek to improve the efficacy and use in the treatment of mesothelioma. Researchers hope to develop photosensitizers that specifically target cancer cells and have more toxic reactions. They also hope to find a more effective means of administering the necessary light which can penetrate tissue and treat larger tumors.^43^

## PROGNOSIS

Prognosis is mainly determined by gender, stage of clinical presentation and the level of completeness of cytoreduction. A significant difference between women and men (13 months vs. 6 months, respectively) (P<0.001) has been reported in literature.^44^ Studies have proved that mean survival has improved by the use of intraperitoneal chemotherapy. Patients who successfully underwent surgical resection had a considerably longer median survival (20 months vs. 4 months, P<0.001) as well as a significantly higher 5-year survival (28% vs. 12%, P<0.001). Multivariate analysis identified that a poorly differentiated tumor grade, failure to undertake surgical resection, advanced age, and male gender were all independent predictors of poorer outcome.^9^

Literature review conclude that the Pericardial and peritoneal MM have worse prognosis (median survival 5e6.9 months) than pleural MM (median survival 7.9e10 months). A recent analysis reported shorter survival for the most peritoneal MM cases.^44^

## CONCLUSION

Malignant Mesothelioma (MM) is a rare but rapidly fatal and aggressive tumor with limited knowledge of its natural history. The diagnosis of peritoneal and Pleural mesothelioma is often delayed, due to a long latent period between onset and symptoms and the common and nonspecific clinical presentation. A considerably high level of index of suspicion must be borne in mind and further studies are required to reach the definitive diagnosis.
